# Surgical management of superior mesenteric artery syndrome in a 16-year-old female: a case report and literature review

**DOI:** 10.1097/RC9.0000000000000582

**Published:** 2026-06-09

**Authors:** Rand Abou Eid, Ibrahim Aljnide, Hadi Salah Aldeen, Ahmad Al-Bitar, Samer Muhsen, Mahmoud Mohamed Dabrha

**Affiliations:** a Faculty of Medicine, Al-Andalus University, Qadmous, Syrian Arab Republic; b Faculty of Medicine, Damascus University, Damascus, Syrian Arab Republic; c Department of Surgery, Damascus Hospital, Damascus, Syrian Arab Republic

**Keywords:** case report, duodenal obstruction, duodenojejunostomy, nutcracker syndrome, superior mesenteric artery syndrome, Wilkie’s syndrome

## Abstract

**Introduction:**

Superior mesenteric artery syndrome (SMAS) is a rare gastrointestinal disorder caused by the compression of the third part of the duodenum between the superior mesenteric artery and the aorta. It typically presents with nonspecific upper gastrointestinal (GI) obstructive symptoms, making diagnosis challenging. This case report illustrates a classic presentation, diagnostic workup, and successful management of SMAS while highlighting a common comorbidity.

**Case presentation:**

A 16-year-old Arab female presented with symptoms of upper GI obstruction. Upper endoscopy revealed extrinsic duodenal compression. An abdominal computed tomography scan confirmed the diagnosis, showing a reduced aortomesenteric angle (12–28°) and distance (6–7 mm). Concurrent compression of the left renal vein (nutcracker syndrome) was also identified. After failed conservative management, the patient successfully underwent a duodenojejunostomy, which was converted to an open approach. Postoperatively, she experienced complete resolution of symptoms and significant weight gain.

**Clinical discussion:**

This case aligns with the typical demographic of SMAS, affecting young, slender females. Diagnosis relies on a high index of suspicion and radiological confirmation of a narrowed aortomesenteric angle and distance. The management follows a stepwise approach, beginning with conservative measures. Surgical intervention, specifically duodenojejunostomy, is highly effective when conservative therapy fails. The coexistence of nutcracker syndrome in this patient underscores the importance of a comprehensive radiological assessment to identify associated vascular compression syndromes.

**Conclusion:**

This report reinforces the importance of precise radiological criteria for diagnosing SMAS and adheres to the standard treatment algorithm. Surgical bypass via duodenojejunostomy resulted in an excellent outcome. Clinicians should maintain a high index of suspicion for SMAS in young patients with chronic upper GI symptoms and be aware of potential associated conditions, like nutcracker syndrome.

## Introduction

Superior mesenteric artery syndrome (SMAS), also referred to as Wilkie’s syndrome or mesenteric clamp syndrome, is a rare and often challenging gastrointestinal (GI) disorder^[^1^]^. It is characterized by the compression of the third portion of the duodenum between the superior mesenteric artery anteriorly and the aorta and spine posteriorly^[^2^]^. This compression typically occurs due to a reduction of the normal aortomesenteric angle, often precipitated by a loss of the intervening retroperitoneal fat pad, which can happen after rapid weight loss or in association with certain spinal deformities^[^1,3^]^. Epidemiologically, SMAS is very uncommon, with a reported incidence ranging from 0.013% to 0.3%, and it predominantly affects adolescents and young adults, particularly females^[^2,4^]^. The clinical presentation is variable but often includes chronic and nonspecific upper GI symptoms such as postprandial epigastric pain, nausea, vomiting, early satiety, and weight loss, which can mimic other more common conditions^[^1,3^]^. Due to its rarity and nonspecific symptoms, diagnosis requires a high index of suspicion and relies on a combination of clinical presentation and radiological confirmation^[^2^]^. Timely diagnosis is crucial, as a delay can lead to serious complications like electrolyte imbalances, gastric perforation, and peritonitis^[^4^]^. Management strategies range from conservative medical treatment to surgical intervention, depending on the severity and response to initial therapy^[^1,3^]^.

HIGHLIGHTS

- Maintain a high index of suspicion for superior mesenteric artery syndrome (SMAS) in young, slender females with chronic upper gastrointestinal obstructive symptoms, as its nonspecific presentation can lead to diagnostic delays and serious complications.
- Adhere to a stepwise management approach, proceeding to duodenojejunostomy when conservative measures fail, and ensure comprehensive radiological assessment to identify associated conditions, such as nutcracker syndrome.

This case report has been presented in line with the SCARE checklist^[^5^]^.

## Case presentation

**Figure 1.**
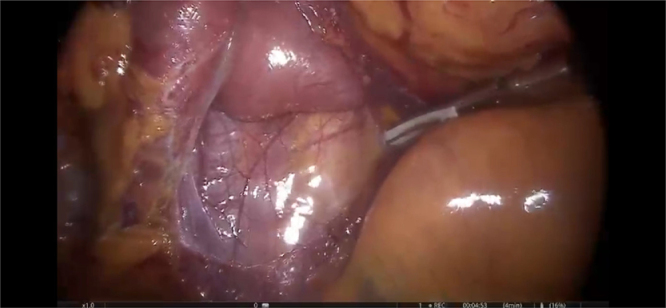
Endoscopy – sequential endoscopic views showing (1) hypotonic stomach with retained gastric fluid.

**Figure 2.**
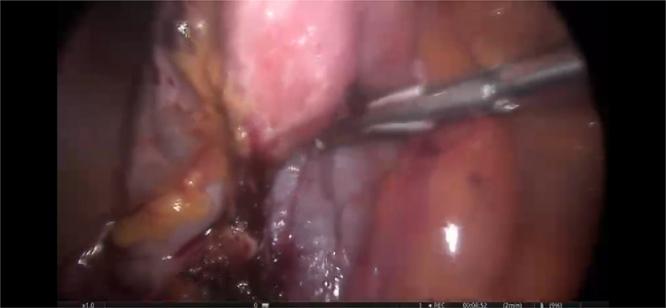
Extrinsic compression at the second part of the duodenum without mucosal abnormality. No ulcers or masses were identified.

A 16-year-old Arab female presented with symptoms indicative of upper GI obstruction. At presentation, her height was 162 cm, weight was 42 kg, and body mass index (BMI) was 16.0 kg/m^2^ (below the 5th percentile for age and sex). She reported an unintentional weight loss of approximately 8 kg over the preceding 3 months. Her diagnostic journey began with an upper endoscopy. The endoscopic examination revealed a hypotonic stomach and significant compression and narrowing at the second and third parts of the duodenum, with no visible ulcer or mass to account for the obstruction. The initial impression was suspicious of an extrinsic compression (Figs 1 and 2).

**Figure 3.**
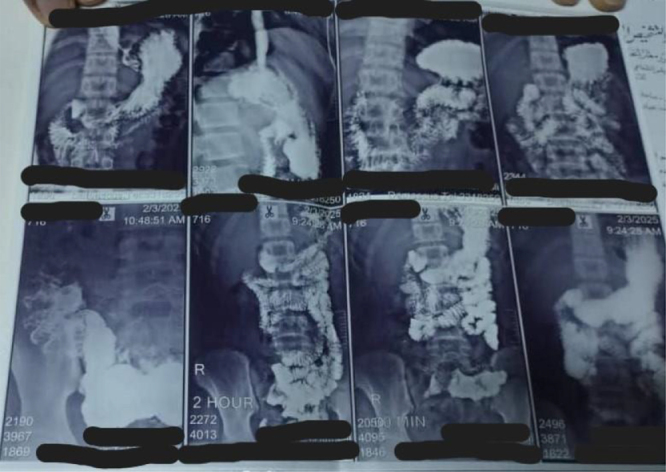
CT scan **–** contrast-enhanced abdominal CT in the coronal plane demonstrating the reduced aortomesenteric angle.

Based on this finding, a computed tomography (CT) scan of the abdomen and pelvis with contrast was recommended and subsequently performed. Multiple CT reports consistently identified the key radiographic signs. These included a significantly reduced aortomesenteric angle, measuring between 12° and 28°, which is well below the normal range of greater than 38–40° (Fig. 3). The aortomesenteric distance was also narrowed to 6–7 mm, compared to a normal distance of more than 8–10 mm. The third part of the duodenum was seen passing through this narrowed space, with associated compression and mild proximal dilation. Additionally, compression of the left renal vein between the aorta and superior mesenteric artery was noted, consistent with nutcracker syndrome, a condition known to coexist with the primary diagnosis.

The collective findings from endoscopy and cross-sectional imaging led to a final diagnosis of SMAS, or Wilkie’s Syndrome, complicated by nutcracker phenomenon.

The patient was initially managed conservatively for 4 weeks. This regimen included nasogastric tube decompression, strict postprandial positioning (left lateral decubitus or knee–chest position), and nutritional support via a nasojejunal tube with continuous elemental formula infusion (1500 kcal/day). Despite these measures, she failed to gain weight (BMI remained 16.1 kg/m^2^) and continued to experience daily postprandial vomiting and epigastric pain. Conservative management was declared a failure due to the absence of clinical improvement and persistent obstructive symptoms after 4 weeks of optimized medical therapy. Given the failure of conservative management to relieve her persistent obstructive symptoms, the decision was made to proceed with surgical intervention.

The patient underwent a planned laparoscopic duodenojejunostomy. The procedure began with mobilization of the second, third, and fourth parts of the duodenum, and a jejunal loop was selected for anastomosis. However, due to technical difficulties encountered during the laparoscopic phase, the decision was made to convert to an open approach through a right paraumbilical incision. The specific technical difficulties included: (1) poor visualization of the retroperitoneal anatomy due to inadequate bowel retraction and lack of sufficient working space; (2) difficulty mobilizing the duodenum off the overlying superior mesenteric artery because of fibrotic adhesions in the aortomesenteric angle; and (3) an inability to safely create a tension-free anastomosis laparoscopically without risking injury to the mesenteric vessels. The selected bowel segments were exteriorized, and a lateral duodenojejunal anastomosis was successfully created using a gastrointestinal anastomosis stapler. A Z-suction drain was placed adjacent to the anastomosis, and the abdomen was closed in layers.

Postoperatively, the patient resumed oral intake on day 5 after a water-soluble contrast study confirmed anastomotic patency. She was discharged on postoperative day 8. Her postoperative course was favorable. At her 2-week and 6-week follow-up appointments, she reported a complete resolution of her obstructive symptoms. She was able to tolerate a regular diet without nausea or vomiting. The surgical incision was well-healed, and the drain had been removed prior to discharge. At discharge, her weight was 44 kg (BMI 16.8 kg/m^2^). She demonstrated steady weight gain, having regained over 80% of the weight she had lost preoperatively. At her 3-month follow-up, her weight was 48 kg (BMI 18.3 kg/m^2^). She remained asymptomatic and had successfully returned to all normal activities, including school and light athletics. The intervention successfully achieved its goal of relieving the duodenal obstruction, with an excellent clinical outcome.

## Discussion

The case of this 16-year-old Arab female exemplifies a classic, yet complex, presentation of SMAS, which aligns with established diagnostic criteria and management pathways described in the literature. This discussion focuses on three key educational takeaways: (1) the critical role of precise radiological measurements in confirming SMAS, (2) the stepwise management algorithm with clear criteria for declaring conservative failure, and (3) the intraoperative decision-making process when converting from laparoscopic to open surgery. Additionally, the notable coexistence with nutcracker syndrome is highlighted.

### Epidemiological and demographic profile

This case is highly consistent with the typical epidemiological profile of SMAS. The patient is a young female in her second decade of life, which is the most commonly affected demographic. Literature reviews note that the median age of patients is 23 years, with a female-to-male predominance ratio of 3:2^[^6^]^. The estimated incidence in the general population is low, ranging from 0.013% to 0.3%^[^6,7^]^, making this a rare diagnosis that requires a high index of suspicion, particularly in young, thin patients with upper GI obstructive symptoms.

### Radiological confirmation

The diagnosis in this case was secured through a combination of clinical suspicion and precise radiological findings, which is the standard of care. The upper endoscopy, which revealed extrinsic compression without an intrinsic mass, appropriately directed the investigation toward cross-sectional imaging. The abdominal CT scan demonstrated the pathognomonic features of SMAS: a significantly reduced aortomesenteric angle of 12–28° and a narrowed aortomesenteric distance of 6–7 mm. These measurements fall squarely within the established diagnostic thresholds for SMAS, where the aortomesenteric angle is typically less than 25° (normal: 38–56°) and the distance is reduced to 2–8 mm (normal: 10–20 mm). A critical educational point is that a narrow SMA angle alone can be found in asymptomatic, low-BMI individuals and is not sufficient for diagnosis; clinical correlation is essential**^[^**8^]^.

### Management algorithm and failure criteria

The management of this patient followed the widely accepted stepwise approach for SMAS. Initial conservative management is always the first-line strategy, with reported success rates of 70–80%^[^6,7^]^. The goal of conservative therapy is to break the vicious cycle of weight loss and increased duodenal compression through nutritional support. In this case, conservative management was continued for 4 weeks and included nasogastric decompression, nasojejunal tube feeds (1500 kcal/day), and postural positioning. The specific criteria used to declare failure were: (1) lack of significant weight gain (BMI increased only from 16.0 to 16.1 kg/m^2^) and (2) persistence of daily postprandial vomiting and epigastric pain. Establishing objective failure criteria is essential to avoid prolonged ineffective medical therapy and to timely refer for surgery. Surgical intervention is required in approximately 20–30% of patients^[^7^]^.

### Surgical technique and lessons from conversion to open surgery

The performed procedure, a duodenojejunostomy, is the most common and successful surgical operation for SMAS, with success rates cited between 80% and 100%^[^6^]^. This procedure relieves the obstruction by creating an anastomosis between the dilated duodenum and the jejunum, effectively bypassing the site of vascular compression. The decision to convert from laparoscopic to open surgery was made due to three specific technical difficulties: poor visualization from inadequate bowel retraction, fibrotic adhesions in the aortomesenteric angle preventing safe duodenal mobilization, and inability to create a tension-free anastomosis laparoscopically. The key educational takeaway is that conversion should not be viewed as a failure but as sound surgical judgment to prevent complications such as vascular injury or anastomotic leak. Surgeons planning laparoscopic duodenojejunostomy should have a low threshold for conversion in the presence of dense adhesions or unfavorable anatomy. The patient’s excellent outcome – complete resolution of symptoms, ability to tolerate a regular diet, and significant weight gain (BMI 18.3 kg/m^2^ at 3 months) – is consistent with the generally positive prognosis reported following timely surgical correction^[^7^]^.

### Coexistence with nutcracker syndrome: clinical implications

A distinctive and educationally important feature of this case is the concomitant diagnosis of nutcracker syndrome (compression of the left renal vein between the aorta and the SMA). This association is well-recognized in the literature, as both conditions stem from the same anatomical narrowing of the aortomesenteric compartment^[^7^]^. The clinical implication is twofold: first, radiologists and clinicians should actively look for left renal vein compression when SMAS is diagnosed; second, the presence of nutcracker syndrome may influence long-term follow-up, as some patients develop hematuria or left flank pain even after duodenal obstruction is relieved. In this patient, no renal symptoms were present at diagnosis or during follow-up, but awareness of this potential comorbidity allows for appropriate counseling and monitoring.

### Comparison with literature

Table 1 summarizes the key features of the presented case compared with the general literature consensus on SMAS in pediatric and adolescent populations.

**Table 1:** Comparison with literature.

Feature	Presented case (16-year-old female)	Literature consensus
Demographics	Adolescent, female	Young adults (median 23), female predominance (3:2)^[^6^]^
Key symptoms	Postprandial pain, nausea, vomiting, weight loss	Nausea, vomiting, epigastric pain, early satiety, weight loss^[^6^]^
Radiologic diagnosis	CT: aortomesenteric angle 12–28°, distance 6–7 mm	CT: angle <25°, distance 2–8 mm^[^7^]^
Co-morbidity	Nutcracker syndrome	Known association with nutcracker syndrome^[^6,7^]^
Management	Failed conservative → Duodenojejunostomy	Conservative first (70–80% success); surgery if fails (80–100% success)^[^6^]^
Outcome	Complete resolution of symptoms, weight gain (BMI 16.0→18.3)	Excellent with appropriate intervention^[^7^]^

## Conclusion

This report reinforces the importance of precise radiological criteria for diagnosing SMAS and adheres to the standard treatment algorithm. Three key lessons emerge for clinicians: (1) objective aortomesenteric measurements on sagittal CT are essential for confirmation; (2) conservative management should be time-limited (e.g., 4 weeks) with clear failure criteria, such as lack of weight gain and persistent symptoms; and (3) conversion from laparoscopic to open duodenojejunostomy is a prudent intraoperative decision when technical difficulties arise, not a complication. Surgical bypass via duodenojejunostomy resulted in an excellent outcome for this patient, with complete symptom resolution and sustained weight gain (BMI 16.0 → 18.3 kg/m^2^ at 3 months). Clinicians should maintain a high index of suspicion for SMAS in young patients with chronic upper GI symptoms and be aware of potential associated conditions, such as nutcracker syndrome.

## Data Availability

Not applicable.
